# High *Sclerotinia sclerotiorum* resistance in rapeseed plant has been achieved by *OsPGIP6*

**DOI:** 10.3389/fpls.2022.970716

**Published:** 2022-09-15

**Authors:** Meng Yin, Rui Wang, Shi Li, Mei Luo, Wei Wei, Maolin Wang, Jun Jiang, Yongjun Lin, Yun Zhao

**Affiliations:** ^1^Key Laboratory of Bio-Resource and Eco-Environment of Ministry of Education, College of Life Sciences, Sichuan University, Chengdu, China; ^2^Institute of Crop Research, Sichuan Academy of Agricultural Sciences, Chengdu, China; ^3^National Key Laboratory of Crop Genetic Improvement and National Centre of Plant Gene Research, Huazhong Agricultural University, Wuhan, China

**Keywords:** *Sclerotinia sclerotiorum*, *Brassica napus*, PGIP, PG inhibitory activity, *Arabidopsis thaliana*

## Abstract

*Sclerotinia sclerotiorum*, a worldwide distributed fungal pathogen, causes serious adverse effects on the yield and seed quality of rapeseed. Polygalacturonase-inhibiting proteins (PGIPs) can protect the cell wall from degradation by pathogen-secreted polygalacturonases (PGs). The present study found several PGIPs from *Oryza sativa*, especially OsPGIP6 and 3 have much higher inhibitory activities to SsPGs than BnPGIP2 from *Brassica napus*. Among them, *OsPGIP1, 4, 6* can significantly elevate the resistance of transgenic Arabidopsis to *S. sclerotiorum*. Subsequently, *OsPGIP1, 3, 4, 6* were subjected to SSR resistance assay in transgenic rapeseed plants. Among which, *OsPGIP6* showed the highest resistance to *S. sclerotiorum*. At 48 h after detached leaves inoculation, the lesion area of OE-OsPGIP6 rapeseed plants is only 17.93% of the non-transgenic line, and 22.17, 21.32, 52.78, 56.47%, compared to OE-BnPGIP2, OE-OsPGIP1, OE-OsPGIP2, OE-OsPGIP4, respectively. Furthermore, the lesion area of OE-OsPGIP6 reached 10.11% compared to WT at 72 hpi. Also, the lesion length on the stem of OE-OsPGIP6 plants was reduced by 36.83% compared to WT. These results reveal that *OsPGIP* family, especially *OsPGIP6*, has a great potential in rapeseed *S. sclerotiorum*-resistance breeding.

## Introduction

*Sclerotinia sclerotiorum* (Lib.) de Bary, one of the worldwide distributed necrotrophic fungal pathogens, can infect more than 400 plant species, including some important economic crops, such as rapeseed, soybean, sunflower, and so on, among which rapeseed (*Brassica napus* L.), as one of the most important oil crops worldwide, deserves great attention (Bolton et al., 2006). Studies show that Sclerotinia stem rot (SSR) caused by *S. sclerotiorum* can lead to severe yield loss,10%−70% yield loss per year, and results in low oil quality due to a decrease in oil content, erucic acid content, glucosinolate, and protein content. The *S. sclerotiorum* attack host during all growth stages especially in the flowering period, causing white watery lesions at the stem, siliques, and leaf, which consequently affect the host physiological activities. For a long time, the obtained breeding materials were still compromised in further agricultural applications (Zhao and Wang, 2004; Liu et al., 2005; Mei et al., 2015). In addition, the pathogenesis mechanisms of SSR still remain elusive. Current research on the pathogenesis of SSR and the crop genetic improvement for SSR resistance mainly focuses on mining major genes associated with innate defense signaling pathways. Rice, as a typical model plant, have gained great attention, since it lives in high temperature and humidity and is susceptible to fungal infection. Therefore, the rice-derived resistance genes may have an important potential in fighting against fungal disease for other plants.

Till now, some breakthroughs have been made in the study of the pathogenicity and effector of *S. sclerotiorum*. Researchers proposed that the acidic environment rather than oxalic acid is the key factor in determining the virulence of *S. sclerotiorum*, namely PH-dependent theory (Liang et al., 2015; Xu et al., 2015, 2018). *S. sclerotiorum* can detoxify reactive oxygen species (ROS) by regulating the “copper ion import/transport” pathway. And the hosts competitively absorb copper ions in the lesion to restrict *S. sclerotiorum* (Dingid et al., 2020). In the face of the Brassicaceae plant's glucosinolate-myrosinase system, *S. sclerotiorum* metabolize isothiocyanates effectively *via* hydrolysis to amines that are not toxic to the fungus (Chen et al., 2020). In addition to the above studies, many other effectors involved in the pathogenicity of *S. sclerotiorum* have also been reported. For example, the SsCP1, a cerato-platanin protein, targeting host PR1 in apoplast facilitates the infection of *S. sclerotiorum* (Yang et al., 2018). The SsITL targets a chloroplast-localized calcium-sensing receptor (CAS) inhibiting SA accumulation during the early stage of infection and contributes to the virulence of *S. sclerotiorum* (Tang et al., 2020). The interaction between *S. sclerotiorum* and hosts remains to be further clarified.

It is well-known that during the early infection stage, *S. sclerotiorum* secretes a series of cell wall degrading enzymes (CWDEs) such as Polygalacturonases (PGs) to facilitate its colonization in the hosts. PGs can degrade the structural polysaccharides between the mesophytic layer and the primary cell wall of plants (Bolton et al., 2006). To fight against the PGs, hosts secrete polygalacturonase-inhibiting proteins (PGIPs) as the defender, which can specifically bind to the active site of PGs to inhibit their activities (Vorwerk et al., 2004; Shanmugam, 2005). Besides, PGIPs can attenuate the degradation of oligosaccharides which are able to elicit defense responses, and thus prolong the protection of the plant against pathogens (Mishra et al., 2012).

*PGIPs* have been widely studied with a transgenic approach in many species including Arabidopsis (Ferrari et al., 2012), rice (Lu et al., 2012; Wang et al., 2015; Feng et al., 2016), wheat (Janni et al., 2013), tobacco (Joubert et al., 2006), rapeseed (HuangFu et al., 2014; Wang et al., 2018), apple (Oelofse et al., 2006), cabbage (Hwang et al., 2010), tomato (Schacht et al., 2011), kiwi (Szankowski et al., 2003), grapevine (Szankowski et al., 2003; Richter et al., 2006; Nguema-Ona et al., 2013; Zhang et al., 2021), mulberry (Hu et al., 2012), and mungbean (Chotechung et al., 2016). The results suggest that *PGIPs* play crucial roles in plant fungi resistance. Therefore, *PGIPs* are considered effective candidate genes against *S. sclerotiorum*. Rice is an important crop as well as a model plant around the world. It is worth noting that rice is often cultivated in high-temperature and high-humidity environments, which makes it easier to confront with fungi. However, few studies have been carried out to discover and identify gene resources in rice that fight against fungal disease for other crops, such as oilseed rape. As for *OsPGIPs, OsPGIP1, OsPGIP2*, and *OsPGIP4* have been identified to be involved in the defense against sheath blight and bacterial leaf streak, respectively, in transgenic plants (Wang et al., 2015; Feng et al., 2016). And another research showed that *OsPGIP2* confers *S. sclerotiorum* resistance in *B. napus* through increased activation of defense mechanisms (Wang et al., 2018). Therefore, researchers have proposed that increasing the expression of *OsPGIPs* by transgenic technology might correspondingly increase the resistance to pathogens in transgenic lines.

In the present study, five *OsPGIPs* (*OsPGIP1, OsPGIP2, OsPGIP3, OsPGIP4, OsPGIP6*) and *BnPGIP2* were functionally identified for their resistance against *S. sclerotiorum* both *in vitro* and *in vivo*. OsPGIPs showed obvious inhibitory activities toward PGs from *S. sclerotiorum*, and remarkably OsPGIP6 and OsPGIP3 exhibited high-level inhibitory activities. The results of pathogenicity assays showed that overexpressing *OsPGIP2, OsPGIP3, OsPGIP4*, and *OsPGIP6* in rapeseed plants conferred significantly elevated resistance to *S. sclerotiorum*, and the *S. sclerotiorum* resistances were consistent with the SsPGs inhibitory activities. Among these genes, *OsPGIP6* showed the highest resistance to *S. sclerotiorum* in both pathogenicity assays and PGIP activity assays, suggesting their potential values in crop breeding for *S. sclerotiorum* resistance, and a batch of high-quality transgenic resistance materials represented by OE-OsPGIP6-3 have been screened in this study.

## Materials and methods

### Prokaryotic expression and western blot analysis

The total genomic DNA of Nipponbare and *B. napus* was used as a template to amplify *OsPGIP1, OsPGIP2, OsPGIP3, OsPGIP4, OsPGIP6*, and *BnPGIP2* (accession numbers: AM180652, AM180653, AM180654, AM180655, NM-001068720, and EU142024, respectively) with specific primers. The PCR-generated fragments were inserted into T-vector and confirmed by sequencing with primers SP6 and T7. Sequence-confirmed clones containing the target genes were digested by corresponding restriction enzymes and, respectively, cloned into prokaryotic expression vector pMAL-c2X.

Positive recombinant plasmids were, respectively, transformed into *E. coli transetta (DE3)*, and then grown at 37°C. When the cultures reached an OD_600_ of 0.5, protein expression was induced by the addition of IPTG to 0.1 mM at 16°C for 20 h. Cells collected by centrifugation at 12,000 rpm for 1 min at 4°C were re-suspended in pre-cooled Hepes buffer (pH 7.0) and then broken by ultrasonication and centrifuged at 12,000 rpm for 10 min at 4°C. The supernatant and sediment were analyzed by SDS-PAGE.

For Western blot analysis, soluble proteins with different OsPGIP-MBP or MBP control were, respectively, transferred to PVDF membranes after SDS-PAGE. Western blot analysis was performed with Anti-MBP Monoclonal Antibody (New England Biolabs, E8032, 1:10,000 working dilution) as the primary antibody and HRP-conjugated murine antibody (1:10,000 working dilution) as the second antibody according to standard protocols.

### SsPGs inhibitory activity *in vitro*

PGs from *S. sclerotiorum* were isolated according to a previous study. PGIP activities were determined by reducing end-group analysis with the 3, 5-Dinitrosalicylic Acid (DNS) method (HuangFu et al., 2014). Ten units of PG from *S. sclerotiorum* (strain Ep-1PNA367) were mixed with different prokaryotically expressed proteins and were then incubated for 30 min at 25°C. Then the mixtures were, respectively, added with polygalacturonic acid (2.5 mg/ml) and incubated for 1 h at 30°C. The experiment was performed in triplicate.

### *OsPGIPs* overexpression vector construction and *Agrobacterium*-mediated transformation

Each PGIP was amplified with specific primers. Sequence-confirmed clones containing the target genes were digested by corresponding restriction enzymes and, respectively, cloned into pCHF3. The positive clones were transformed into the *Agrobacterium tumefaciens* strain *GV3101* by electroporation. Subsequently, all the constructs were introduced into Arabidopsis (ecotype Columbia-0) and rapeseed (Westar) by *Agrobacterium*-mediated transformation (Chotechung et al., 2016). The transformation procedures were carried out as previously described (Janni et al., 2013). Subsequently, PCR analysis was performed to determine the presence of the target genes in transgenic plants with the specific primers 35S-F and 35S-R.

### Gene expression analysis

The total RNAs of the transgenic plants were extracted and reverse-transcribed as described previously (Lu et al., 2012). Reverse transcription PCR (RT-PCR), as well as quantitative real-time PCR (qRT-PCR), were performed according to the same reference. The relative expression levels were determined using $2_{\text{T}}^{-\Delta \Delta \text{C}}$ method (Schmittgen and Livak, 2001).

### Leaf inoculation and detached stem inoculation

The PCR-positive transformed plants and untransformed controls were grown in the field to the 9–10 leaf stage. And pathogenicity assay was performed with detached leaves as previously described (HuangFu et al., 2014). The sixth-eighth leaf of each transgenic plant was removed for detached leaf inoculation with *S. sclerotiorum* in a greenhouse. Maintaining a relatively high humidity is a key factor in this experiment. The leaf lesions were taken pictures and measured at 48 h and 72 h post-inoculation (hpi). Each treatment was performed with three independent biological replicates. And lesion sizes were measured using Image J. Normally growing rapeseed were screened for stem inoculation of *S. scleroterum*. Agar discs (8 mm in diameter) were excised from the edges of growing fungal colonies, which were then affixed onto rapeseed stems 50 cm from the ground. Disease severity was assessed by measuring lesion length per pathogen infection spot at 120 hpi. Inoculation, observation, and disease resistance were only determined for plants located in the center of blocks to avoid marginal effects.

## Results

### OsPGIP6 and OsPGIP3 showed high inhibitory activities against SsPGs

**Figure 1:**
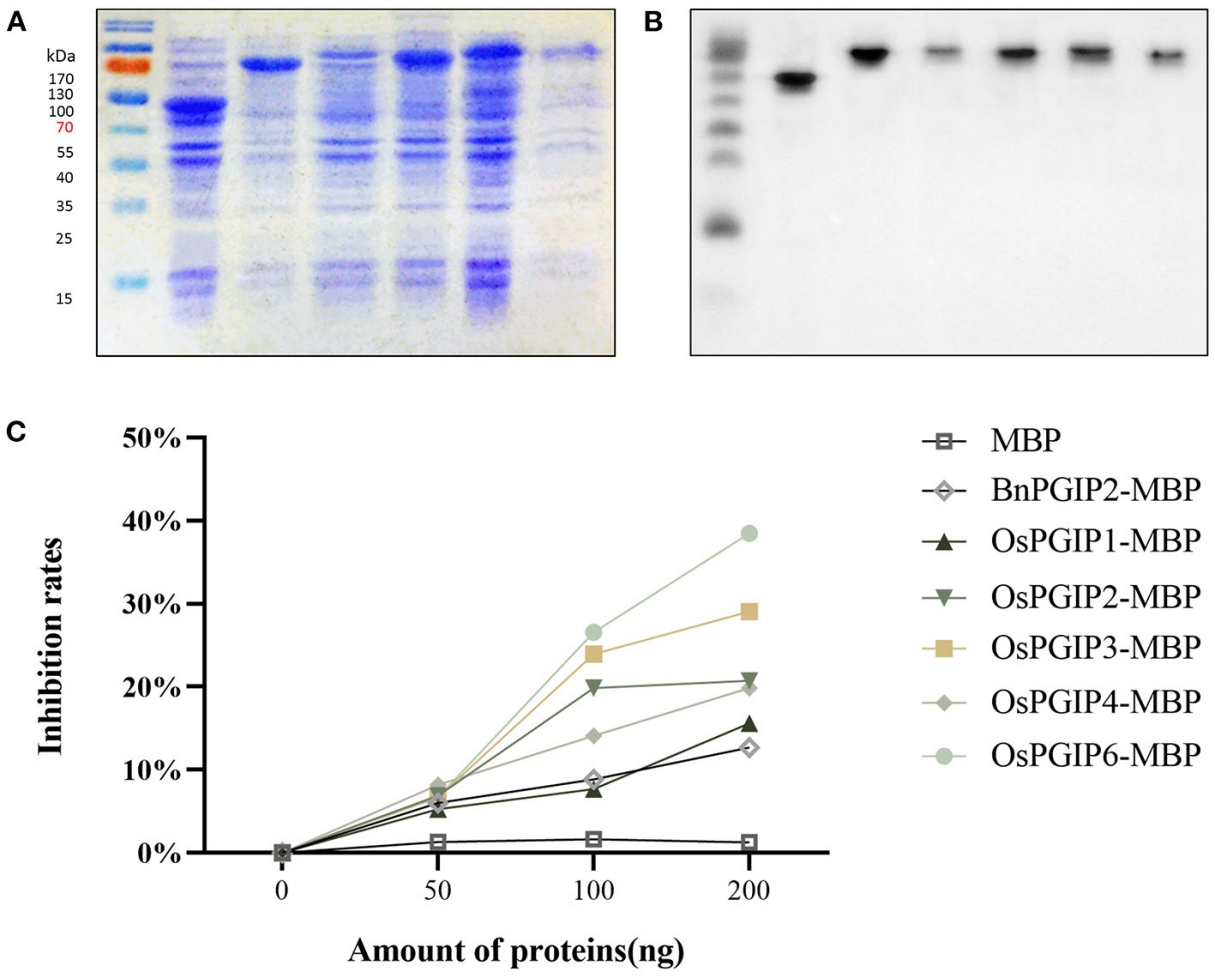
OsPGIPs show PG inhibitory activity of *S. sclerotiorum in vitro*. **(A)** Supernatant protein of prokaryotic expression. Expression of OsPGIPx in E. coli. M, marker; Lane 1, 20 h after IPTG inducement with MBP; Lane 2–6, 20 h after IPTG inducement with OsPGIP-MBP (Lane 2: OsPGIP1-MBP; Lane 3: OsPGIP2-MBP; Lane 4: OsPGIP3-MBP; Lane 5: OsPGIP4-MBP; Lane 6: OsPGIP6-MBP). **(B)** The corresponding immune signals of target proteins in **(A)** were generated by Western blot. Western blot was performed with Anti-MBP Monoclonal Antibody (1:10,000 working dilution) as primary antibody and HRP-conjugated murine antibody (1:10,000 working dilution) as second antibody. **(C)** OsPGIP activity assays. Variation of *S. sclerotiorum* PG activity with increasing amounts (0–200 ng) of different prokaryotically expressed proteins. Ten units of PG and 2.5 mg/ml polygalacturonic acid were used in the assays. *In vitro* inhibition of enzyme activity by purified PGIP protein equal the reduction percentage of D-GA in the reaction.

In order to characterize inhibitory activities of OsPGIPs including OsPGIP1, OsPGIP2, OsPGIP3, OsPGIP4, and OsPGIP6 to PGs from *S. sclerotiorum*, the OsPGIPs:MBP in-frame gene fusions were, respectively, expressed in *E.coli* Rosetta (DE3) cell (Figure 1). The SDS-PAGE results suggested that highly active fusion protein could be obtained in the supernatant (Figure 1A). Western blot assay further proved the target proteins were successfully obtained (Figure 1B).

The PGs isolated from *S. sclerotiorum* were used for OsPGIPs activity assay. *In vitro* activity assays showed that SsPGs inhibitory activities of different PGIPs are diverse (Figure 1C). PG inhibitory rate of OsPGIP6 is high up to 26.56%, followed by OsPGIP3, OsPGIP2, OsPGIP4, OsPGIP1, and BnPGIP2 in 100 ng purified protein treatment. The PGs inhibitory rate of each PGIP has increased as the amount of purified protein did. When elevated to 200 ng, the inhibition rates of different PGIPs were increased to 38.48, 29.06, 20.75, 19.86, 15.59, and 12.67%, respectively. The results indicate that the five OsPGIPs can significantly reduce the activity of PGs hydrolyzing polygalacturonic acid *in vitro*. Notably, OsPGIP6 performed outstandingly.

### OEPGIP4, OEPGIP1, OEPGIP2, and OEPGIP6 exhibited significantly elevated resistance to *S. sclerotiorum* in Arabidopsis

**Figure 2:**
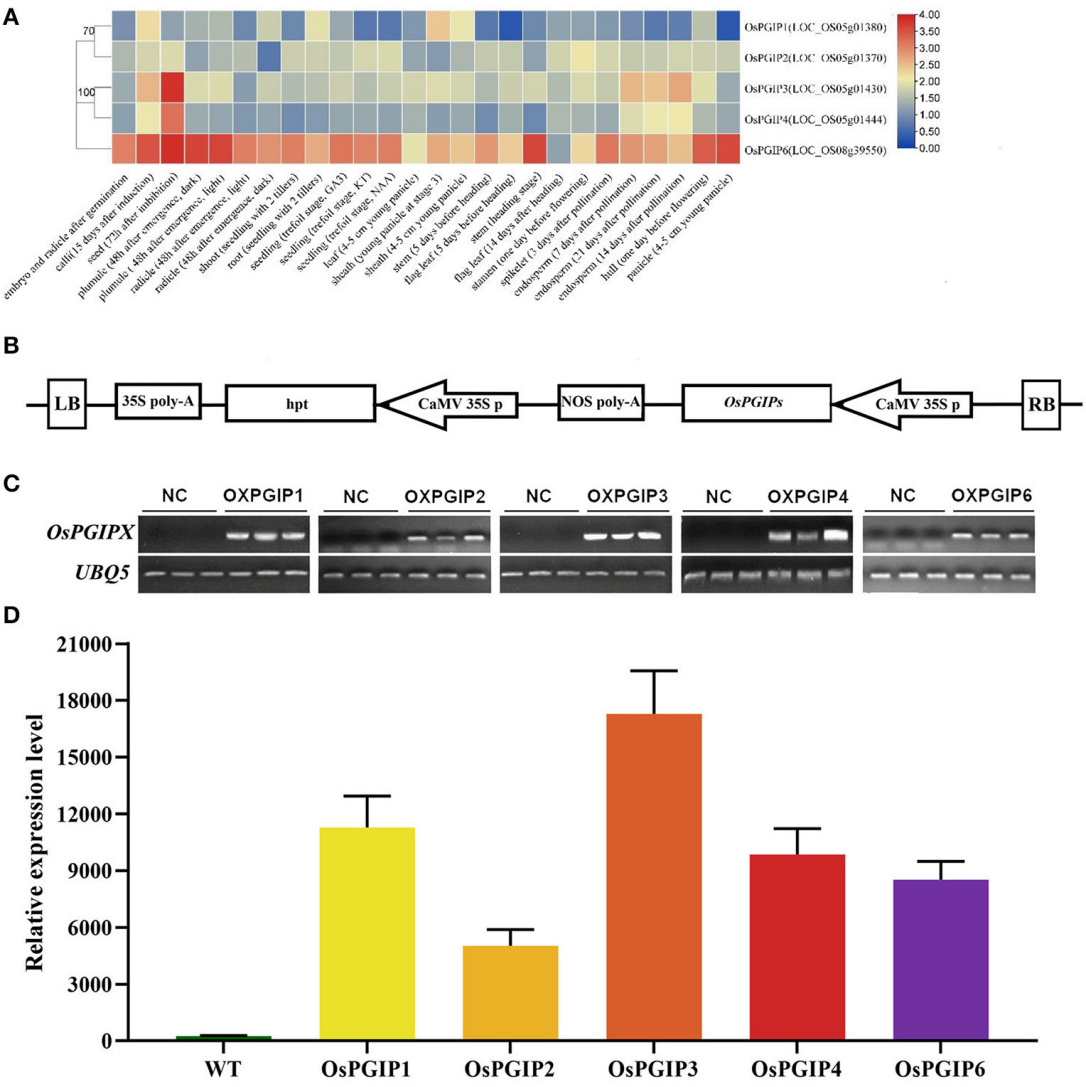
The expression analysis of OsPGIPs in rice and transgenic Arabidopsis plants. **(A)** The expression of OsPGIPs in various tissues and organs throughout the entire life cycle of rice. The color scale (representing |lg signal values|) is shown on the right. **(B)** Schemes of recombinant plasmids for overexpression. The expression level of each target gene was analyzed by RT-PCR **(C)** and qRT-PCR **(D)** with three independent transgenic lines. NC, negative control, Error bars indicate SE based on three independent biological replicates.

Subsequently, the five OsPGIPs were subjected to in vivo resistance assay. Their expression levels in various tissues and organs throughout the entire life cycle of rice were shown in Figure 2A. The construct containing *OsPGIPs* under the control of *35S* promoter were, respectively, transformed into *Arabidopsis thaliana* (ecotype Columbia-0) (Figure 2B). Three overexpression transgenic lines of each OsPGIP were obtained after semi-quantitative PCR (Figure 2C) and quantitative real-time PCR (qRT-PCR) (Figure 2D) identification. The target gene was expressed at high levels in all transgenic lines, and we performed inoculation assay in these OE-*OsPGIPs* lines.

**Figure 3:**
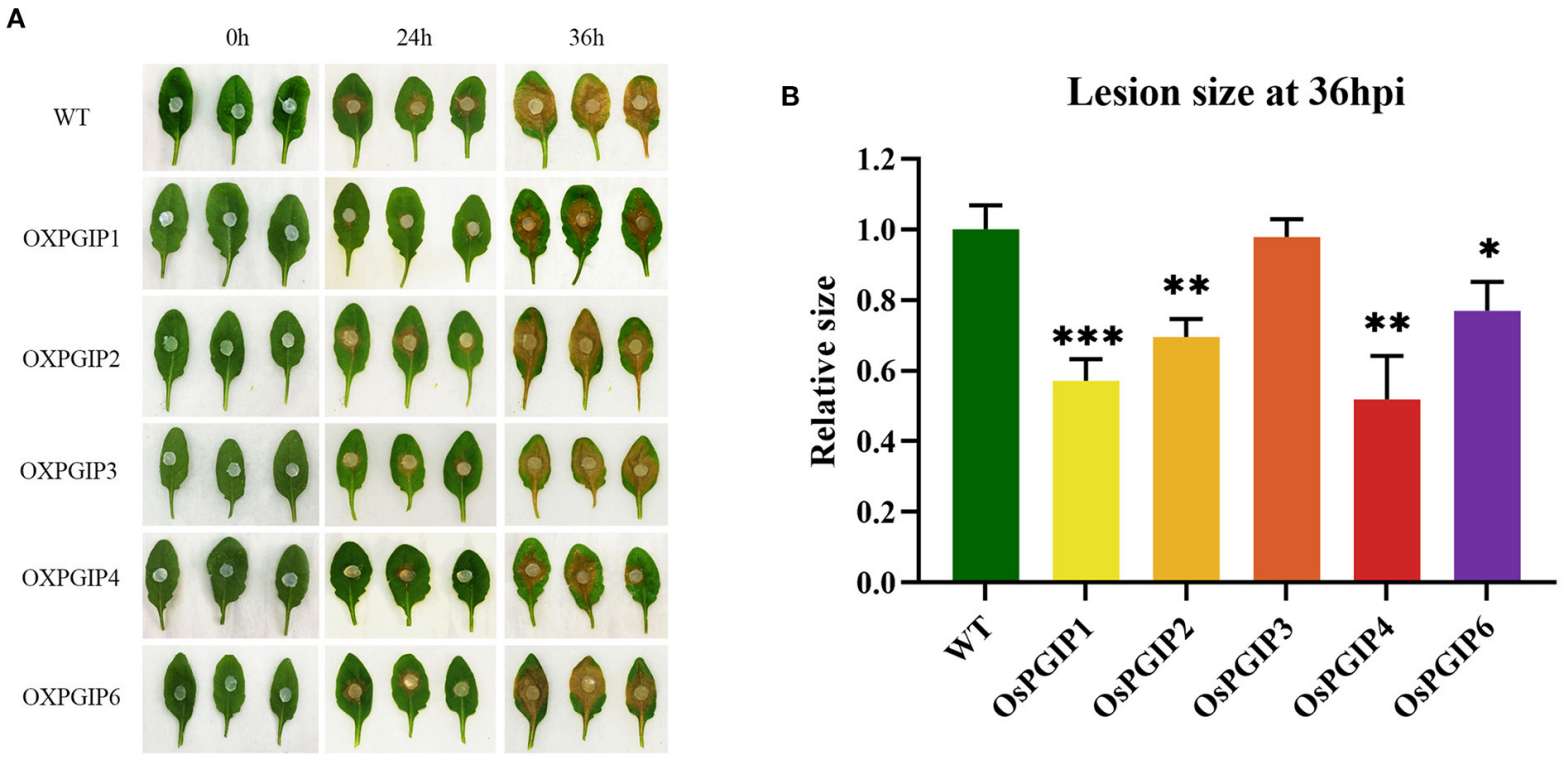
*S. sclerotiorum* resistance assay in *OsPGIPx* transgenic Arabidopsis. **(A)** Phenotypes of pathogenicity assays on detached leaves from different transgenic plants at 0 h, 24 h and 36 h after inoculation with *S. sclerotiorum*. **(B)** Statistical analysis of relative lesion sizes on the inoculated leaves at 36 hpi. Significant differences were showed by: ***P < 0.001, **P < 0.01, *P < 0.05. Error bars indicate SE based on three independent biological replicates.

To determine the *in vivo* functions of the five *OsPGIPs* in *S. sclerotiorum*-plant interaction, the leaves detached from *OsPGIP* transgenic plants (OE-OsPGIPs) were, respectively, subjected to pathogenicity assay (Figure 3). 36 h after the inoculation of *S. sclerotiorum*, the infection phenotypes of the leaves were obviously diverse among different transgenic plants. The leaves of OE-OsPGIP1 and OE-OsPGIP4 showed the highest resistance to this pathogen; OE-OsPGIP2 and OE-OsPGIP6 also exhibited resistance, but it was lower than that of OE-OsPGIP1 and OE-OsPGIP4; while OE-OsPGIP3 showed no obviously elevated resistance. Furthermore, the sizes of the spreading lesions on the leaves from *OsPGIPx* transgenic plants (Figure 3B) indicated that their resistance to *S. sclerotiorum* followed the order of OE-OsPGIP4, OE-OsPGIP1, OE-OsPGIP2, OE-OsPGIP6, OE-OsPGIP3. Meanwhile, OE-OsPGIP4, OE-OsPGIP1, OE-OsPGIP2, and OE-OsPGIP6 showed significantly elevated resistance compared to the non-transgenic plants. These results also suggested that the *S. sclerotiorum* resistance of the transgenic plants overexpressing different *OsPGIPs* was in accordance with their inhibitory activities to PGs from this pathogen.

### *OsPGIPs* overexpression conferred resistance to *S. sclerotiorum* in transgenic rapeseed plants

**Figure 4:**
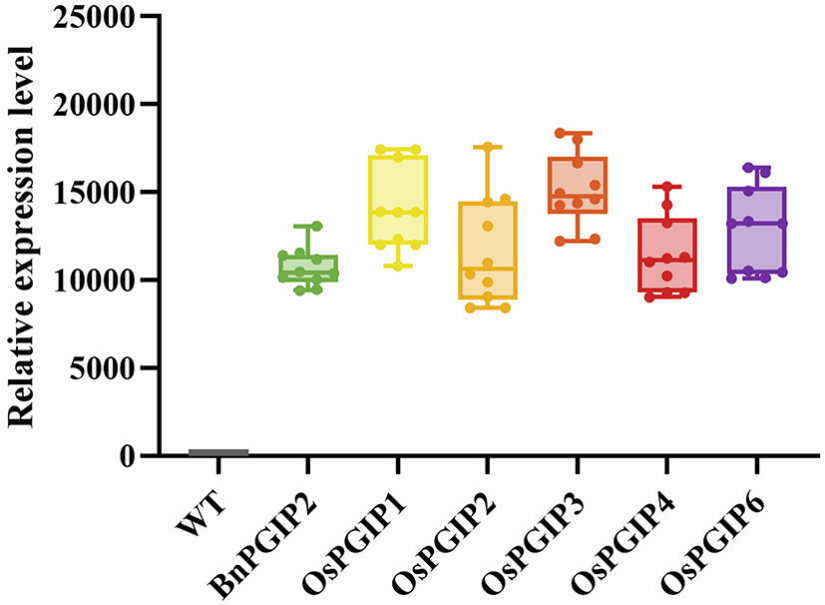
Relative expression of *OsPGIPx* in transgenic rapeseed plants. Ten individual plants in T_0_ generation of each *PGIP* transgenic rapeseeds were subjected to expression analysis.

Quantitative real-time PCR (qRT-PCR) was used to detect the expression levels of target genes in transgenic rapeseed plants. Ten independent biological replicates in T_0_ generation of each *PGIP* transgenic rapeseeds were subjected to expression analysis (Figure 4). Based on the results, five individual transgenic families of each *PGIP* with representative expression levels were selected for *S. sclerotiorum* resistance assay, their seed yields per plant were shown in Supplementary Figure 1.

**Figure 5:**
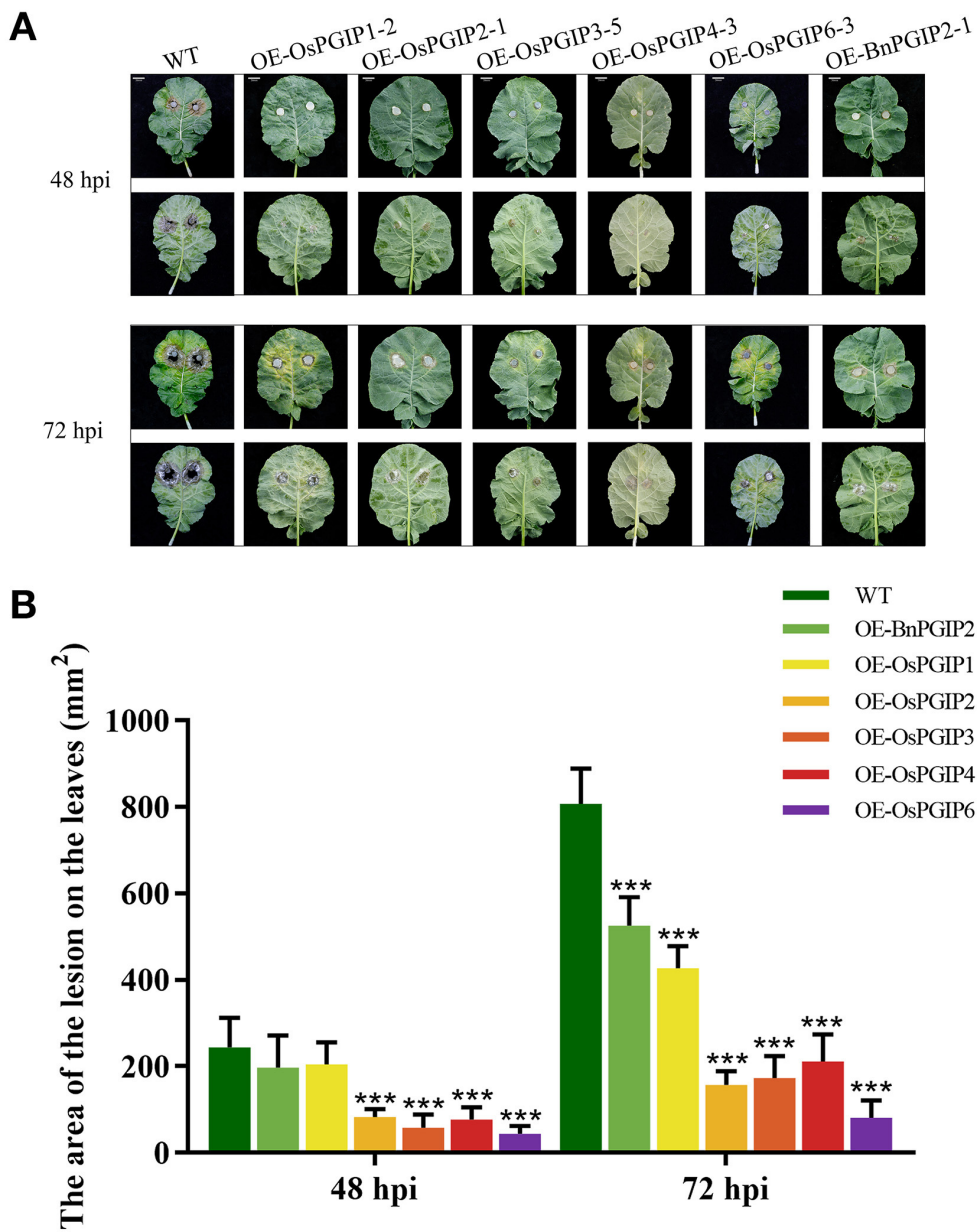
*OsPGIPx* overexpression conferred resistance to *S. sclerotiorum* in detached leaves inoculation. **(A)** Detached leaves of overexpression lines and non-transgenic line inoculated with 10-mm agar plugs of *S. sclerotiorum* hyphae at 22°C. Images were taken at 48 and 72 h post-inoculation (hpi). Scale bars are 20 mm. **(B)** Lesion on detached leaves at 48 and 72 hpi. Significant differences were showed by: ***P < 0.001. Error bars indicate SE based on five independent biological replicates.

**Figure 6:**
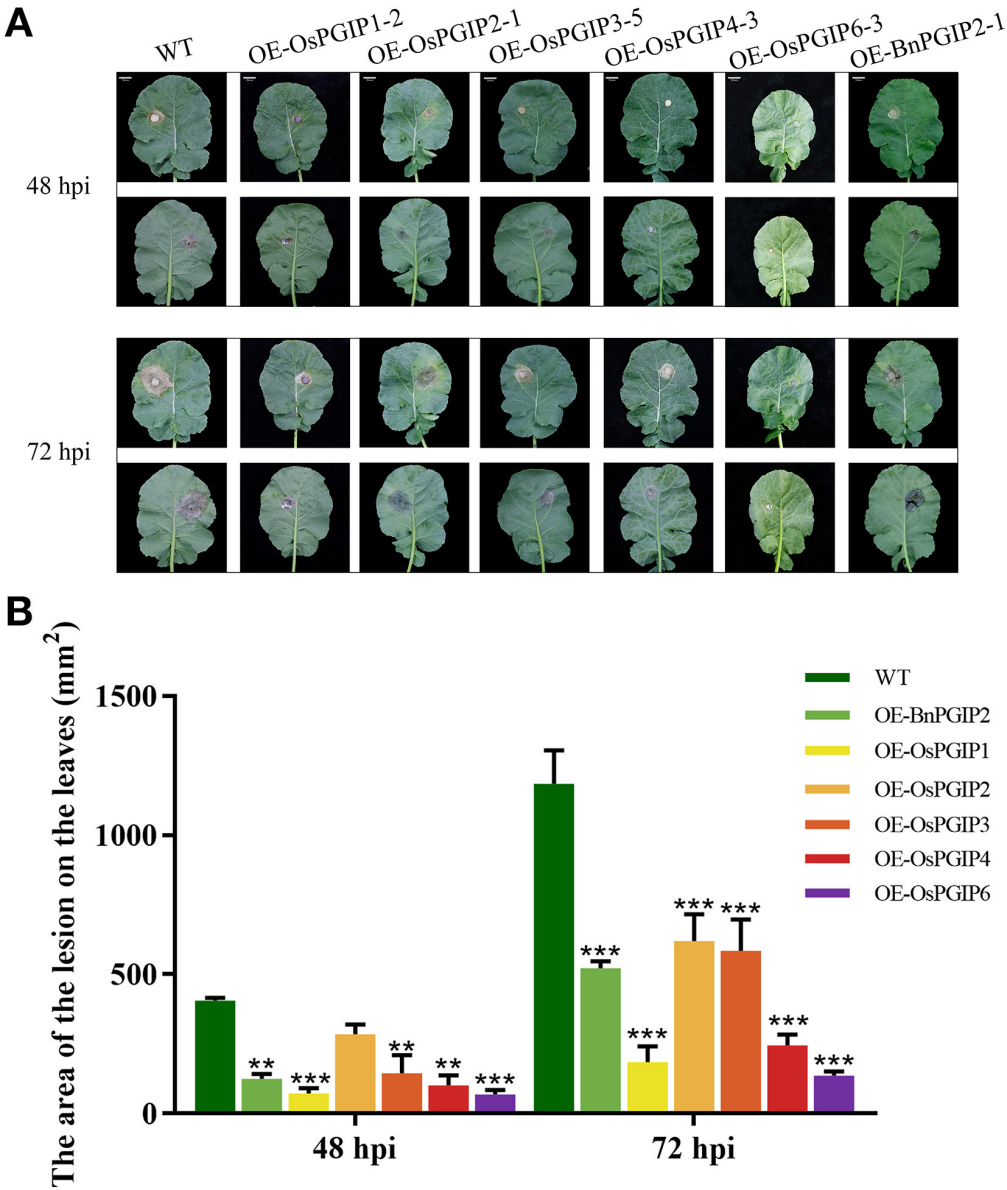
*OsPGIPx* overexpression conferred resistance to *S. sclerotiorum* in Living leaves inoculation. **(A)** Living leaves of overexpression lines and non-transgenic line inoculated with 10-mm agar plugs of *S. sclerotiorum* hyphae at 22°C. Lesion on living leaves at 48 and 72 hpi. Scale bars are 20 mm. **(B)** Lesion on living leaves at 48 and 72 hpi. Significant differences were showed by: ***P < 0.001, **P < 0.01. Error bars indicate SE based on five independent biological replicates.

**Figure 7:**
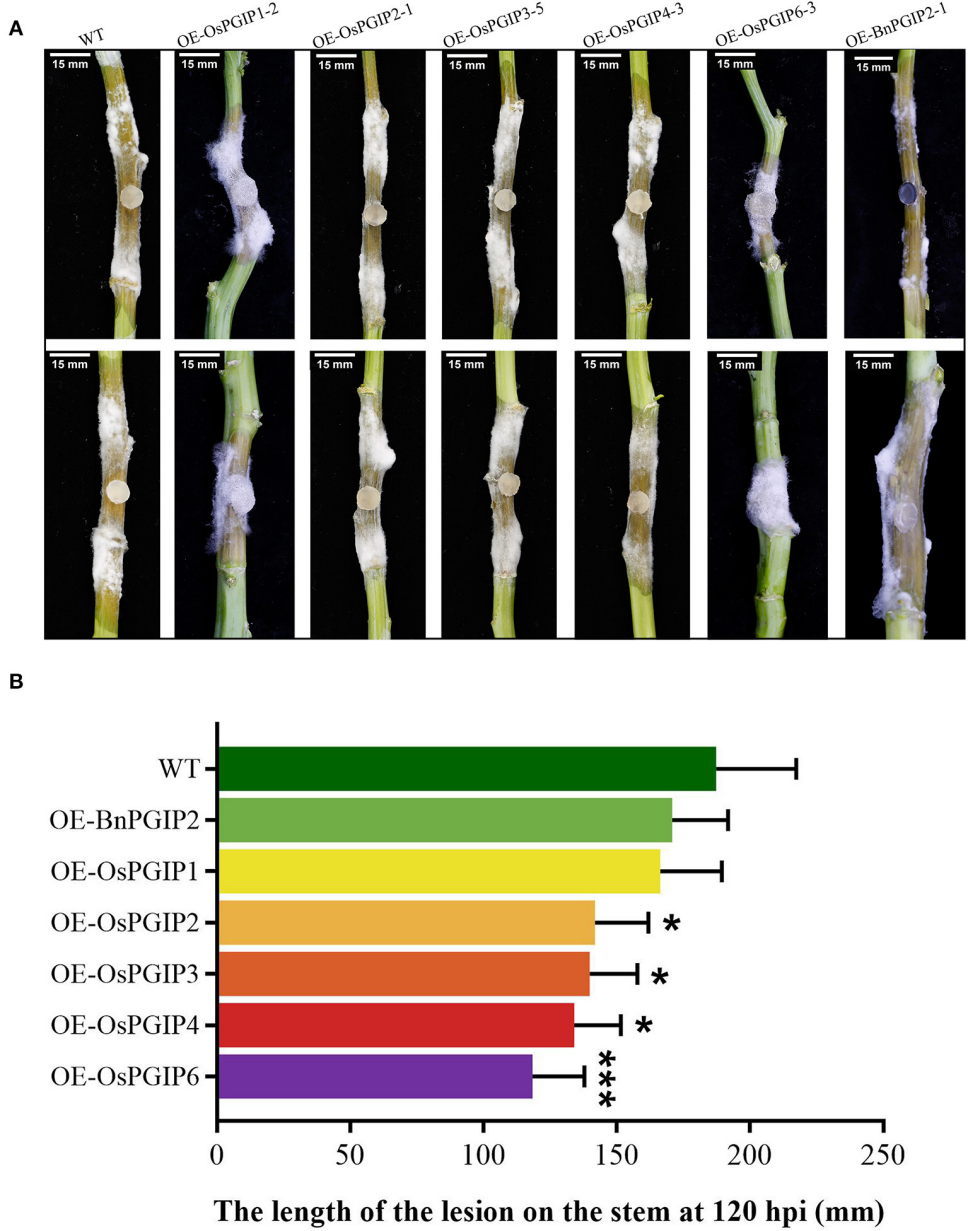
*OsPGIPx* overexpression conferred resistance to *S. sclerotiorum* in detached stem inoculation. **(A)** Stems of transgenic lines and non-transgenic line 120 h after inoculation with *S. sclerotiorum*. Scale bars are 15 mm. **(B)** Lesion on stem at 120 hpi. Significant differences were showed by: ***P < 0.001, *P < 0.05. Error bars indicate SE based on five independent biological replicates.

The resistance of *S. sclerotiorum* was identified by leaf inoculation at the 9–10 leaf stage and detached stem inoculation at the flowering stage. The results told that 48 and 72 h after inoculation with *S. sclerotiorum*, the transgenic plant lesion areas were smaller than that of the wild-type material significantly. In the detached leaf inoculation, the transgenic lines, OE-OsPGIP6 and OE-OsPGIP3, showed a greatly improved resistance to SSR, especially OE-OsPGIP6. The lesion area of transgenic lines OE-OsPGIP6 and OE-OsPGIP3 are 43.63 and 57.64 mm^2^, respectively, while the non-transgenic line presented a lesion area of 243.36 mm^2^ (Figure 5) at 48 hpi. At 72 hpi, the lesion area of OE-OsPGIP6 was even reduced to 10.11% compared to the non-transgenic lines. In addition, some other transgenic strains also showed enhanced *S. sclerotiorum* resistance by delaying pathogen infection. When living leaves were inoculated with *S. sclerotiorum*, the transgenic lines, OE-OsPGIP6 and OE-OsPGIP1, showed the best resistance to SSR, with a lesion area reduction of 88.52 and 84.38% at 72 hpi (Figure 6). In the green-pod stage, the resistance of *S. sclerotiorum* was further identified by detached stem inoculation. The experimental results showed that the resistances of the detached stems from OE-OsPGIP2, OE-OsPGIP3, OE-OsPGIP4, and OE-OsPGIP6 to *S. sclerotiorum* were significantly higher than that of the wild-type after 120 h inoculation with *S. sclerotiorum*, and the lesion length caused by *S. sclerotiorum* infection was significantly lower, with a reduction of 24.27, 25.33, 28.47, and 36.83%, respectively (Figure 7).

## Discussion

In nature, rice live in high temperature and high humidity environments which are more susceptible to fungi. Considering that a dozen *PGIPs* have been found in *B. napus* while few have resistance to *S. sclerotiorum*, we speculated that the *PGIPs* family from rice may have more ideal resistance potential to fighting against SSR (Bashi et al., 2013; HuangFu et al., 2014). *BnPGIP2* was selected for its superior performance in *BnPGIPs* against *S. sclerotiorum* (Wang et al., 2021). Our study confirms this hypothesis, which shows that *OsPGIPs* have better *S. sclerotiorum* resistance than *BnPGIP2* when introduced in *B. napus*, providing a new implication for rapeseed *S. sclerotiorum*-resistance breeding.

Although *OsPGIP* has been reported for its resistance to *S. sclerotiorum*, the candidate gene was only confined to *OsPGIP2* (Wang et al., 2018). In this study, the performance of the living leaves of *OsPGIP2*-overexpressing rapeseed plants was not ideal compared to other *OsPGIPs*, which may be related to the functional specificity of different *PGIPs*. Therefore, the resistance gene screened by a detached test could not meet the needs of the application. Here, we found that rapeseeds overexpressing *OsPGIP6* performed much better than *OsPGIP2* in *S. sclerotiorum* invasion. Moreover, *OsPGIP6*-transgenic plants showed stable high resistances to *S. sclerotiorum* in different inoculation experiments. Hence, *OsPGIP6* is a valuable *S. sclerotiorum*-resistance gene both in theory and applications.

The present study demonstrates that the *PGIPs* family from rice can effectively elevate the resistance of transgenic plants to SSR. By *in vitro* assays, the PG inhibitory activities of OsPGIPs were all evaluated. Subsequently, these *OsPGIPs* were overexpressed in Arabidopsis for pathogenicity assays. Pathogenicity assays of transgenic plants showed that *OsPGIP1, OsPGIP2, OsPGIP4*, and *OsPGIP6* could significantly improve the resistance of Arabidopsis to *S. sclerotiorum*.

The PGIP activity assays showed that the inhibitory activities of different OsPGIPs to *S. sclerotiorum* PGs were widely divergent. OsPGIP6 and OsPGIP3 showed strong inhibitory activities, while OsPGIP1 showed relatively low inhibitory activity. The results are also consistent with the finding of previous reports, PGIPs are diverse in their inhibition specificities and potentials. Hence, it can be inferred that despite the conservation of *PGIPs* family across different species during evolution, each *PGIP* within the family is specific, especially in the domain involved in the interaction with PGs. In addition, five *OsPGIPs* in Arabidopsis and rapeseed showed inconsistent resistances to *S. sclerotiorum*, which may be caused by the complex polyploidy genome of *B. napus*. As an allotetraploid plant, a more complicated defense system could further improve or alter the effects of resistance genes.

The PGIP-PG interaction is a classic immune evolutionary system model for analyzing the resistant-protein and pathogenic factors. Pathogens produced varieties of PGs to enhance aggressivity, while the hosts have evolved corresponding resistant proteins to minimize harmlessness. Therefore, *PGIPs* are diverse in their inhibition specificities and potentials. Combined with the results, it can be inferred that *OsPGIP6* has a high potential in preventing *S. sclerotiorum* from degrading the plant cell wall. The specific molecular mechanism needs to be elucidated in further study.

The results of living leaves pathogenicity assays showed that the *S. sclerotiorum* resistance of *OsPGIP2* transgenic plants was obviously lower than that of *OsPGIP4* transgenic plants, while the PG-inhibitory activity of OsPGIP2 was almost the same as that of OsPGIP4. This phenotype might arise from the less elevation of expression levels of the target gene in the *OsPGIP2-*overexpressing line compared with the *OsPGIP4*-overexpressing line. Our previous report also showed that the resistance of transgenic plants is related to the expression levels of *PGIP* (Wang et al., 2015). Since the potential PG-inhibitory ability of OsPGIP2 is like that of OsPGIP4, it can be inferred that if the expression level of *OsPGIP2* in transgenic plants is further elevated, the resistance of these plants will be further improved.

In this study, *S. sclerotiorum*-resistant rapeseed plants were obtained by introducing the rice-derived resistance genes, *OsPGIPs*, into *B. napus*, proved by *in vitro* and *in vivo* pathogenicity assays. Different *OsPGIPs* have diverse potential in *S. sclerotiorum* resistance. *OsPGIP6*-overexperssing lines showed vastly lesion reduction compared to the non-transgenic line, also other *OsPGIPs*- and *BnPGIP*- transgenic lines. Overall, our results show that *OsPGIPs* could provide substantial *S. sclerotiorum* resistance in rapeseeds and facilitate SSR-resistance crop breeding.

## Data availability statement

The original contributions presented in the study are included in the article/Supplementary material, further inquiries can be directed to the corresponding author/s.

## Author contributions

YZ, YL, RW, MY, SL, JJ, WW, and MW contributed to design of the study. MY and SL performed the data analysis and completed the original draft. JJ provided partial resources. All authors contributed to manuscript revision, read, and approved the submitted version.

## Funding

This work was supported by the National Key Research and Development Program of China (2016YFD0100202), the National Key Research and Development Plan (2018YFD0100501), Sichuan Province (Grants 2021YJ0296, 2021YFYZ0018, and 2022ZDZX0015), and People's Republic of China. This research was also supported by the Fundamental Research Funds for the Central Universities (SCU2019D013).

## Conflict of interest

The authors declare that the research was conducted in the absence of any commercial or financial relationships that could be construed as a potential conflict of interest.

## Publisher's note

All claims expressed in this article are solely those of the authors and do not necessarily represent those of their affiliated organizations, or those of the publisher, the editors and the reviewers. Any product that may be evaluated in this article, or claim that may be made by its manufacturer, is not guaranteed or endorsed by the publisher.
