# Supplementary figures and images for: High Sclerotinia sclerotiorum resistance in rapeseed plant has been achieved by OsPGIP6
Source: Front Plant Sci. 2022 Sep 15;13:970716. doi: 10.3389/fpls.2022.970716 (PMC9524022; doi:10.3389/fpls.2022.970716)

*Supplementary Material*

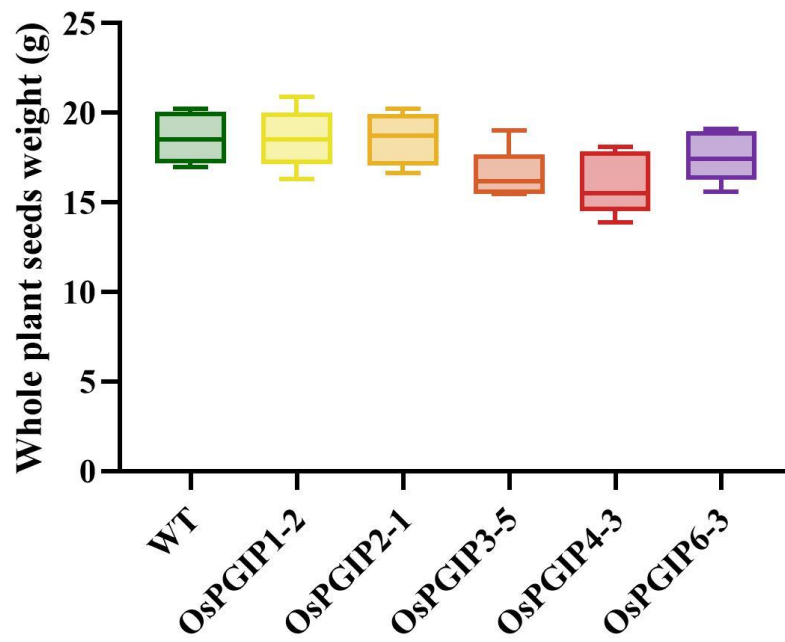

**Figure S1.** Seed yield of individual rapeseed lines.

Supplement: Supplementary file 1 [file Image_1.pdf]
